# Duration of symptom control following intratympanic dexamethasone injections in Meniere’s disease

**DOI:** 10.1007/s00405-022-07368-w

**Published:** 2022-04-10

**Authors:** Aidan Hilton, Alison McClelland, Rhona McCallum, Georgios Kontorinis

**Affiliations:** 1 Faculty of Medicine, University of Glasgow, Glasgow, UK; 2 Department of Otolaryngology-Head and Neck Surgery, Queen Elizabeth University Hospital, 1345 Govan Road, Glasgow, G51 4TF UK

**Keywords:** Corticosteroids, Dizziness, Injection, Meniere’s disease

## Abstract

**Purpose:**

Intratympanic (IT) injections of corticosteroids have emerged as a non-ablative alternative to gentamicin in the management of refractory Meniere’s disease. However, currently, the duration of the symptom control achieved via intratympanic corticosteroids is under reported.

**Methods:**

We retrospectively reviewed the notes of all patients who underwent IT injections of dexamethasone for the treatment of definite Meniere’s disease at a single tertiary referral university centre over a 6-year period. We included demographic information, the number of procedures patients required, duration of symptom-control achieved (time interval between repeat IT injections), and the presence of co-morbidities, with a focus on the presence of autoimmune disease.

**Results:**

We identified 27 patients who underwent a total of 42 procedures; 23/27 (85.2%) patients demonstrated clinical response with a median period of symptom control of 14.5 months (range 1–64, IQR 10.25). The median longest asymptomatic period per patient was 19 months (range 11–64, IQR: 18). Interestingly, all patients with autoimmune disease (7/27) demonstrated a clinical response; autoimmune disease was found to be a statistically significant predictor of response to treatment (*p* = 0.002). In patients who received repeated treatment following disease relapse, there was no difference in duration of symptom-control achieved.

**Conclusions:**

IT steroids can provide an effective alternative to gentamicin ablation. Symptom control is achieved for a median of 14.5 months, and treatment can be repeated with no loss of efficacy. Those patients who have an underlying autoimmune co-morbidity are more likely to demonstrate a clinical response to therapy, which may provide insight into the underlying pathophysiology of Meniere’s disease.

## Introduction

Meniere’s disease (MD) is a chronic, potentially debilitating disorder of the inner ear characterised by episodic bouts of sudden onset vertigo, which can last from minutes to hours, accompanied by sensorineural hearing loss (SNHL), tinnitus and sensations of aural fullness [1, 2].

Intratympanic injections (IT) of Gentamicin has well reported efficacy in reducing vertigo symptoms in patients with refractory MD [3, 4]. However, despite greater selectivity for vestibular toxicity over cochlear toxicity, the risk of hearing loss as well as the radical and irreversible nature of such treatment still limits the use of IT gentamicin [4–6]. IT injections of corticosteroids, chiefly methyl prednisolone or dexamethasone, represent a non-ototoxic, non-ablative alternative to gentamicin. Patel et al*.,* where the first to perform a randomised double-blind trial comparing the reduction in frequency of vertigo attacks achieved by gentamicin vs methylprednisolone, finding them to be equally effective [5]. Subsequent similar studies have been performed leading to a recent meta-analysis that again supported the use of corticosteroids as an alternative to gentamicin [7].

Due to its non-ablative nature, IT steroid injections have become a popular treatment modality attracting research interest; however, the duration of symptom control achieved using IT corticosteroids is, to our knowledge, currently under-reported. Such information would help provide evidence to support the long-term use of IT steroids, allow patients to be counselled more effectively when discussing the efficacy of the procedure, and determine how frequently repeated injections are required for the management of MD. Therefore, the primary objective of this study was to determine the duration of symptom control after IT dexamethasone injection for patients with MD.

## Methods

### Basic settings and patient selection

We carried out a retrospective case series in a tertiary, university hospital setting. The project was approved as a retrospective audit. Caldicott’s guardian approval was also obtained.

We reviewed the medical notes of patients who received IT injections of dexamethasone for MD at a single centre over a 6-year period. We only included patients who had confirmed diagnosis of definite MD as per AAO–HNS 2015 guidelines [8]. These patients were identified through the senior’s authors audit database. Patients were included whether they had unilateral or bilateral disease. Patients with MD who received gentamicin injections as opposed to dexamethasone were excluded.

All included patients had at least one baseline vestibular assessment, including six-canal video-head-impulse-test to ensure vestibular function (presence of vestibular responses from the semi-circular canals) prior to any interventions; this is standard protocol in our institution.

### Procedure

Patients received two intratympanic injections of 3.3 mg/mL dexamethasone steroid 1–2 weeks apart as per standard local protocol; the same protocol applied to the primary as well as the repeat injections. All procedures were performed as day cases at a single centre. We have chosen dexamethasone due to its efficacy as per our experience and its well-tolerated nature by the patient.

Local anaesthetic was administered via lidocaine-soaked gauze strips to the tympanic membrane applied for 5–10 min. Under microscopic vision a 22 Gauge needle was passed through the ear canal and was used to puncture the tympanic membrane twice. The first opening in the tympanic membrane acted as a pressure release (to prevent opening of the Eustachian tube) and then the steroid was administered through the second puncture. Steroid was injected until there was observed overflow via the initial tympanic membrane puncture. Patients were then asked to lie on their back with their head turned towards the contralateral side for 30 min, and to avoid swallowing as much as possible. This also helped to minimise steroid moving through the Eustachian tube, increasing the absorption of steroid from the middle ear into the inner.

Non-response to treatment was defined as no or poor control of their vertiginous symptoms as per patients’ feedback, while response was defined as complete or good control of the patients’ vertiginous symptoms, described as complete resolution of vertigo attacks, or significantly less frequent or severe attacks, as per patients’ feedback. If MD symptoms returned at any points patients were scheduled for another set of injections.

Non-responders were offered either further medical treatment, IT gentamicin therapy or further surgical management, based on a combination of personal preference, symptom severity and past treatments. The details of this further treatment were not recorded in this retrospective review, as it was beyond this project’s aims.

### Outcomes and variables

The following variables were collected for analysis:

Patient characteristics collected for analysis included age, gender, baseline audiometry, presence of unilateral or bilateral disease, years since diagnosis of MD, and presence of any autoimmune co-morbidities.

For outcome assessment we used data from the following: date of first procedure and any subsequent procedures, follow-up time, whether symptom control was achieved, and the duration of symptom control.

The indication for repeat injections was based on the patients’ feedback. If patients reported recurrence of debilitating vertigo attacks, further IT dexamethasone injections were offered. No additional treatment methods were used given the previous good response to IT steroids.

Pure-tone audiometry (PTA) average threshold was calculated based on the threshold at 0.5, 1, 2, and 3 kHz as per AAO–HNS guidelines for studies on MD. Pure tone average of these frequencies was used as a baseline assessment of hearing assessment as part of standard clinical work-up. Given the hearing-protective nature of steroids, the retrospective nature of our study (patients were assessed at different times, so they did not all receive post-injection hearing tests at similar time points) but mainly our primary focus on control of the vestibular symptoms, we did not include any end-point hearing assessment.

Follow-up time was defined as the time period between the first injection to the end of study date (01/10/2020).

Our primary outcome was the duration of symptom control achieved following IT dexamethasone injection. We defined the duration of symptom control as the number of months a patient was asymptomatic of vertigo attacks following the described course of two IT dexamethasone injections. This was either the period between injection and the end of the study period if a patient did not have any further vertigo attacks or if patients had a vertigo attack, the time between sets of steroid injections. For logistical reasons, the time between injections was recorded instead of the time between attacks—this assumed that the waiting times between attacks and intervention were consistent.

Secondary outcomes measures were: (1) the longest asymptomatic period each patient achieved following treatment, (2) the evaluation of the effect of repeated injections on the duration of symptom control achieved; (3) predictive factors for response to treatment, including demographic factors and baseline PTA and (4) whether the presence of autoimmune co-morbidities have influenced response to treatment and duration of response.

### Analysis

We used Minitab Statistical Software for the statistical analysis. Medians, ranges, and interquartile ranges (IQR) as well as means and standard deviations were calculated for all desired outcomes. Following initial analysis, the data was deemed non-parametric due to small sample size. Two proportion testing was used to determine whether any population proportions differed between groups. Mann–Whitney *U* testing was performed as a non-parametric analogue to *t*-testing; comparing two medians to against each other. Kruskal–Wallis was similarly used to compare more than two medians as opposed to ANOVA testing between multiple means.

The level of significance was set at 0.05.

## Results

### Procedural response and symptom-control duration

We identified 27 patients with definite Meniere’s disease who met the inclusion criteria that received 42 sets of IT dexamethasone injections; 23 patients (85.2%) achieved control of their symptoms following the procedure (Table 1). The response group underwent a total of 38 procedures, receiving an average of 1.65 procedures per patient (range 1–4 procedures). Median follow-up time in the response group was 19 months (range 11–82 months). Eleven out of 23 patients required a repeat injection at some point (47.8%) (Table 1).

**Table 1:** Individual patient characteristics and timelines of responses to intratympanic dexamethasone

Patient number	Age	Gender	Clinical response	No. of procedures	Duration of symptom control per procedure (months)				Longest asymptomatic period (months)	Follow-up time (months)
					1st	2nd	3rd	4th		
1	75	Female	Yes	1	19				19	19
2	57	Female	Yes	1	12				12	12
3	61	Female	Yes	1	12				12	12
4	63	Female	Yes	1	12				12	12
5	40	Female	Yes	1	11				11	11
6	73	Female	Yes	1	39				39	39
7	63	Female	Yes	1	16				16	16
8	51	Female	Yes	3	4	6	45		45	56
9	62	Female	Yes	2	4	17			17	21
10	44	Female	Yes	4	9	19	9	46	46	82
11	79	Female	Yes	2	14	34			34	15
12	33	Female	Yes	2	14	2			14	49
13	36	Female	Yes	1	21				21	21
14	65	Male	Yes	1	19				19	19
15	50	Male	Yes	1	19				19	19
16	70	Male	Yes	1	19				19	19
17	61	Male	Yes	1	15				15	15
18	33	Male	Yes	2	5	14			14	19
19	53	Male	Yes	2	10	32			32	42
20	58	Male	Yes	2	12	64			64	76
21	69	Male	Yes	2	16	1			17	18
22	48	Male	Yes	2	25	8			25	33
23	46	Male	Yes	3	33	32	12		33	77
24	67	Female	No	1	0				0	
25	36	Female	No	1	0				0	
26	45	Female	No	1	0				0	
27	46	Male	No	1	0				0	

In the present study 23 out of 27 patients responded to IT Dexamethasone and went on to have a total of 38 procedures as per individual clinical requirements. Of those who responded, 52% (12/23) of patients only required one set of injections, 35% (8/23) required 2 sets, 9% (2/23) required 3 sets, and 4% (1/23) of patients required 4 sets for symptom control. The median duration of symptom control was 14.5 months (range 1–64), and the median longest asymptomatic period was 19 months (range 11–64)

The male: female ratio was 11:16 with a median age 57 years (range 33–79 years, IQR: 18.5) (Tables 1 and 2).

**Table 2:** Period of symptom control by procedure

Procedure	Number of procedures	Duration of symptom control (months)—median (range, IQR)
First	23	14 (4–39, 7.5)
Second	11	17 (1–64, 25)
Third	3	12 (9–45, 18)
Fourth	1	46

Procedure numbers include those who went on to have subsequent injections. Kruskal–Wallis test showed no statistical significance between the duration of response per procedure (*p* = 0.450)

Across all 38 procedures that led to clinical response, the median duration of symptom control was 14.5 months (range 1–64 months, IQR: 10.25). For each of the 23 patients who responded, their longest asymptomatic period following IT injection was a median of 19 months (range 11–64 months, IQR: 18).

### Duration of symptom control per subsequent procedure

To determine if there was a cumulative effect or decreased response to repeated treatment, we compared the duration of symptom control achieved per procedure for the patients who required further intervention.

**Fig. 1:**
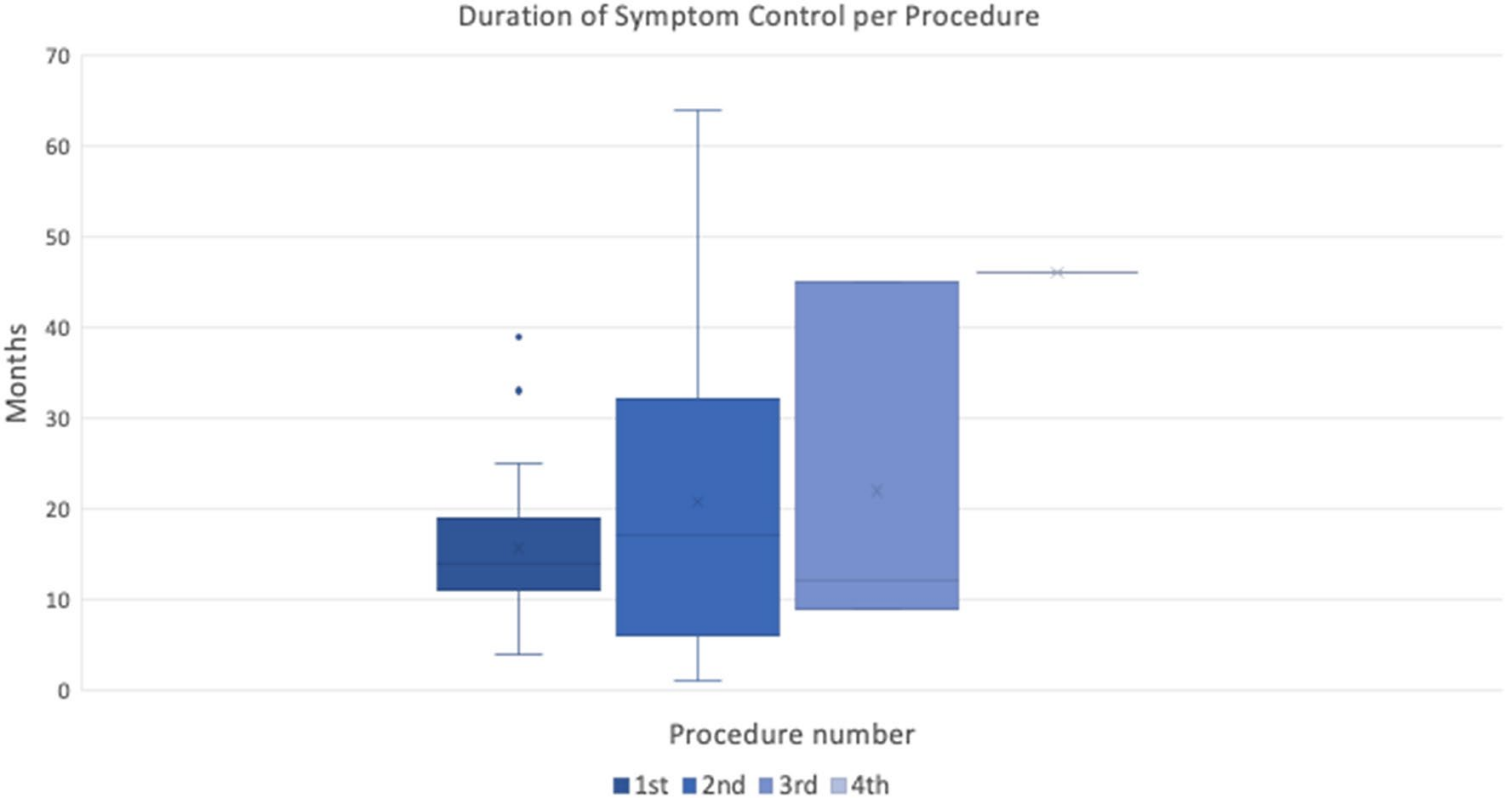
Box and Whiskers plot for the asymptomatic period (months) following intratympanic dexamethasone, comparing the response period from the first procedure to the second, third, and fourth procedures for those patients that underwent further treatment. There was no significant difference in the response duration between procedures

This revealed that there was no statistically significant difference (*p* = 0.450) between the duration of symptom control achieved by patients depending on whether they received their one, two, three or four sets of injections (Fig. 1, Table 2).

### Responders vs non-responders

Despite the small size of the non-responders group, we still compared these patients with the responders groups to check for any predictive factors that may have influenced response. There was no statistical significance between the response group and non-response group when comparing median age (*p* = 0.322), years since diagnosis (*p* = 0.918), or baseline audiometry (0.172) (Table 3). Gender was not a predictor of response to treatment as there was no difference in proportion between females and males in the response or non-response groups (*p* = 0.441), neither was unilateral vs bilateral disease (*p* = 0.741).

**Table 3:** Comparative characteristics of the response vs no response groups

Characteristic	All patients	Responders	Non-responders	*p* values
Number of patients	27	23	4	
Median age (range, IQR)	57 (33–79, 18.5)	58 (33–79, 17)	45.5 (36–67, 8.5)	0.322
Female sex (%)	16 (59)	13 (57)	3 (75)	0.441
Male sex (%)	11 (41)	10 (43)	1 (25)	
Background of autoimmune disease (%)	7 (26)	7 (30)	0	0.002
No background of autoimmune disease (%)	20 (74)	16 (70)	4 (100)	
Unilateral disease (%)	22 (100)	19 (83)	3 (75)	0.741
Bilateral disease (%)	5 (100)	4 (17)	1 (25)	
Median years since diagnosis (range, IQR)	4 (1–36, 4)	4.5 (1–36, 4.75)	4 (3–13, 2.5)	0.918
Median baseline Audio dB (range, IQR)	44 (6.25–80, 36)	40 (6.25–80, 36)	56 (40–71.25, 21.9)	0.172

Neither age, sex, unilateral vs bilateral disease, years since diagnosis or baseline audiometry were predictors of clinical response to treatment. The presence of an autoimmune co-morbidity was identified as a predictor of response

The presence of autoimmune disease was found to be a significant predictor of response to treatment (*p* = 0.002) as all patients with background of autoimmune disease responded to treatment (7/27 patients, 7/23 responders).

### Autoimmune group vs non-autoimmune group

As above, all patients who had an autoimmune co-morbidity (7/27 patients) showed a response to treatment, which was found to be a significant (*p* = 0.002) as a predictor of response to treatment (Table 4).

**Table 4:** Seven patients identified as having autoimmune co-morbidities

Patient number	Autoimmune co-morbidity	Number of procedures	Duration of symptom control per procedure (months)				Longest asymptomatic period (months)
			1st	2nd	3rd	4th	
5	Pernicious Anaemia	1	11				11
12	Polychondritis	2	14	2			14
13	Alopecia	1	21				21
17	Ankylosing Spondylitis	1	15				15
20	Coeliac	2	12	64			64
21	Psoriasis	2	16	1			16
23	Polyarthritis	3	33	32	12		33

Despite being a predictor of response, the presence of an autoimmune co-morbidity did not lead to a difference in the duration of response to IT dexamethasone per procedure or the longest asymptomatic period

To investigate differences in duration of response rate patients were divided into an autoimmune group (7/23 responders) and a non-autoimmune group (16/23 responders).

There were no statistically significant differences between the autoimmune and non-autoimmune groups with regards to age (*p* = 1.000), baseline audiometry (*p* = 0.404) or years since diagnosis (*p* = 0.333).

The median duration of symptom control for procedures performed on the autoimmune group (12 sets of injections across 7 patients) was 14.5 months (range 1–64) vs the non-autoimmune group (26 procedures across 16 patients was 14 months (range 2–46) which was not significant (*p* = 0.867).

The median longest asymptomatic period following treatment for the autoimmune group was 16 months (range 11–64) vs 19 months (range 12–46) for the autoimmune group which was not statistically significant (*p* = 0.841).

**Fig. 2:**
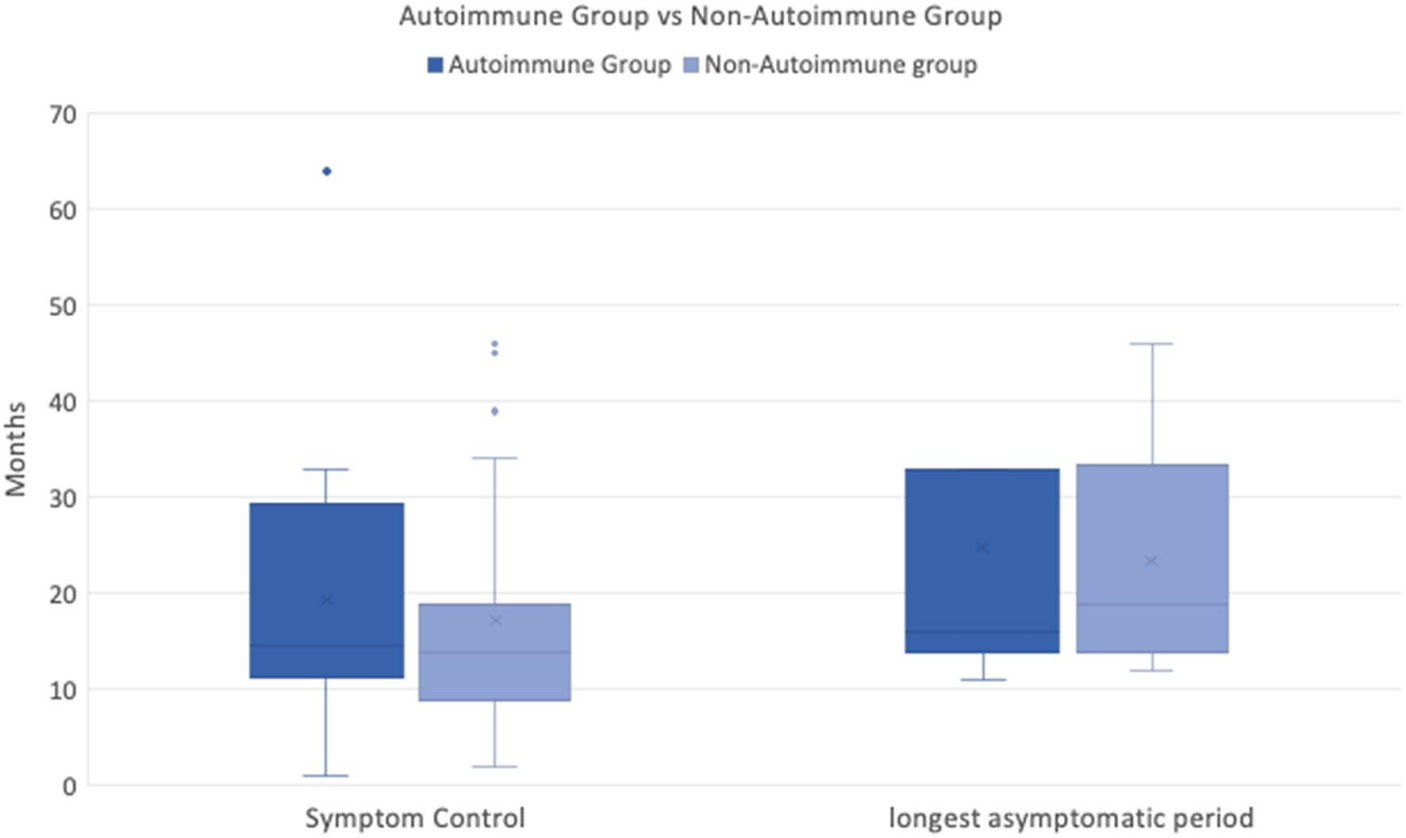
Box and Whiskers comparison of the Autoimmune Group vs the non-autoimmune Group following intratympanic dexamethasone injections. There are 7 patients in the autoimmune group who received 12 total sets of injections per individual clinical requirements. The median duration of symptom control was 14.5 months and the median longest asymptomatic period per patient was 16 months. There are 16 patients in the non-autoimmune group who received a total of 26 sets of injections. The median duration of symptom control was 14 months and the median longest asymptomatic period per patient was 19 months

Therefore, despite being a predictor of response, the presence of an autoimmune co-morbidity did not lead to a difference in the duration of response to IT dexamethasone per procedure or the longest asymptomatic period (Fig. 2).

It is worth mentioning that due to the nature of the study, the precise immune-modulating regime that each patient was on during the examined period was not available. However, as per the medical notes, no changes in any regime were noted for any of the patients in the autoimmune group during the study. Of note, the diagnosis of an autoimmune disease was set by expert rheumatological review in all cases.

## Discussion

### Main outcomes and IT steroid benefits

Previous studies have demonstrated the comparable efficacy of IT injections of dexamethasone or methyl prednisolone vs gentamicin in reducing the frequency of vertigo attacks in MD [5, 7]. However, the length of time that such IT injections can control the patients’ symptoms has barely been examined. Our study showed control of vestibular symptoms with IT dexamethasone injections for a median of 14.5 months with patients reporting symptom control even up to 5 years. Our analysis suggests that neither age, gender, baseline audiometry function, bilaterality or unilaterality of the disease or years since diagnosis affected the duration of symptom control. The median duration of symptom control and the median asymptomatic period were both calculated so that a comparison could be made on an individual patient level as well as a procedural one. Interestingly, we also showed good response in patients with MD and known autoimmune background with seven out of seven responders; this finding is further discussed below.

What is also really important is that the efficacy of IT steroid injections does not seem to change with time, following repeat injections. Indeed, there was no difference between the duration of symptom control with repeated procedures. This suggests steroid injections can continue to be used on a wide range of patients with MD per patient’s requirements and preferences, and that progression to more invasive or radical surgery may not always be warranted. However, the number of patients who received three sets of injections and those who received four sets are extremely low, which greatly affects the power of our statistical interpretation. A longer study with more patients requiring repeat procedures would be needed to confirm whether the efficacy of IT steroids is truly consistent with repeated use. The presented should help with decision making and counselling the symptomatic patient with MD.

### Current knowledge

Gentamicin has been a key treatment in the management of refractory MD; however, possible hearing loss has remained a contentious side effect of treatment, while the radical nature of the treatment can be a decision-making factor against such therapy. A Cochrane review of gentamicin including two studies found that while gentamicin was effective, one of the studies reported a 25% increase in hearing loss (four patients) [4]. Given this risk of hearing loss, coupled with consistently reported increasing prevalence of bilateral MD with increasing disease duration, the non-ablative nature of steroids makes it an appealing alternative [9, 10]. The only known randomised clinical trial comparing IT steroids (methyl prednisolone) with gentamicin confirms the efficacy of IT steroids in dealing with MD [5]. The authors of that study also showed that approximately 50% of their patient needed repeat injections [5]; in our series this was the case in 48% of the enrolled patients, which is in accordance with what is known. The current study adds to the existing knowledge the duration that IT dexamethasone can control the vestibular symptoms for, which is missing from the literature (14.5 month average).

Similarly, to previous report [5], we showed good response to IT steroid injections in patients with MD. While the exact mechanism is still unknown, it is presumed that corticosteroids exhibit their effect via glucocorticoid immune suppression decreasing inflammation within the inner ear. This theory is supported by the potential role of autoimmunity within the development of MD [11]. Studies have demonstrated higher prevalence’s of autoimmune diseases, especially autoimmune arthritis, in patients with MD compared to the general population; likewise, patients with autoimmune disease are more likely to have MD [12–14]. Interestingly, however, an animal study found that IT steroids instead caused an initial rise in inflammatory cytokines as opposed to reduced, and that the steroids may instead work via mineralocorticoid receptors by upregulating genes of ion homeostasis altering endolymph concentrations [1]. The underlying physiology of the response to steroids is interesting but beyond the purpose of this work.

Due to the uncertainty of the role of the immune system in the pathophysiology of MD, we identified patients who had autoimmune co-morbidities and investigate as to whether they responded differently to other patients. As all seven patients identified as having an autoimmune co-morbidity demonstrated a clinical response, it is possible that the presence of an autoimmune co-morbidities could be a predictor of clinical response; the patient sample might be small but still in agreement with previous reports [12–15]. This may suggest that there is indeed an autoimmune component to MD; however, why patients with other autoimmune co-morbidities are more likely to respond to steroid treatment is currently unclear.

### Type of steroid

Dexamethasone was chosen over methyl prednisolone primarily for primarily logistical reasons, as it is widely available in our centre. However, dexamethasone is also advantageous, because it is not associated with stinging pain patients experience with methyl prednisolone injections. Indeed, the pain associated with methyl prednisolone was commented in the randomised control trial of steroids vs gentamicin by Patel et al*.* [5]. For this reason, dexamethasone may be more practical both in clinical trials and for patient experience of the procedure, especially if repeat injections are required. However, recent review showed that while both types of steroids as well as gentamicin are efficient for IT treatments, methyl prednisolone might be a better option for long-term management [16]. Again, determining the ideal type of steroid was beyond the aim of the present work.

### Limitations and strengths

The retrospective nature and the associated risk of patient selection bias are the main limitations of our study. Furthermore, due to the relatively rare nature of MD the patient numbers were low leading to non-parametric data and analysis. Furthermore, we did not monitor post-injection hearing thresholds, mostly because we steroids are believed to be hearing-protective rather than damaging, particularly when compared to gentamicin [4–6]. In addition, as per our methods, patients had their hearing assessed at irregular time intervals following the injections; this fact would have introduced added bias. We also recognise that a control group of placebo or gentamicin would be preferable; however, level 1 evidence has already been published [5]. Of note, our main scope was to determine for how long the injections provide adequate symptom-control and not just to show if they are effective, which has already been reported [7]. Finally, we recognise that only four patients did not respond to the treatment; thus it is a very small group; however, we felt that a direct comparison could highlight factors potentially linked to response/non-response (such as autoimmune background).

On the other hand, the present study took into account several factors, which were included in our statistical analysis, while at the same time, we present a variable but still long follow-ups of patients with MD treated with IT steroid injections. The standardised protocols of IT steroid administration as well as the novelty and rarity of such study adds to the power of our results. Giving a straight answer to the question for how long IT steroids can control the MD vertiginous symptoms but also highlighting that the positive impact of the injections does not weaken with repeated injections carry significant clinical information.

## Conclusions

IT dexamethoasone injections provide an effective alternative to patients with refractory MD with remaining hearing function that do not wish to undergo ablation of labyrinth via gentamicin injections. This retrospective analysis found the average efficacy of IT dexamethasone for MD patients to be for 14.5 months and can be repeated without any loss of its efficacy. Furthermore, patients who have an autoimmune comorbidity are more likely to respond to steroid injections, making them an attractive treatment option in this cohort of patients.

## Data Availability

Upon request with limitations due to anonymity and ethical considerations.
